# Multimodal Imaging of Unilateral Benign Yellow Dot Maculopathy

**DOI:** 10.1177/24741264241260489

**Published:** 2024-06-16

**Authors:** Michael Balas, Jovi Wong, Parnian Arjmand

**Affiliations:** 1 Temerty Faculty of Medicine, University of Toronto, Toronto, ON, Canada; 2 Department of Ophthalmology and Vision Sciences, University of Toronto, Toronto, ON, Canada; 3 Mississauga Retina Institute, Toronto, ON, Canada

**Keywords:** Unilateral yellow dot maculopathy, macular dystrophy, ancillary imaging

## Abstract

**Purpose:** To describe the multimodal imaging findings associated with benign yellow dot maculopathy. **Methods:** A case report was analyzed. **Results:** A healthy 42-year-old White man was evaluated after several weeks of photopsias and an inferior retinal tear in the right eye. The tear was treated with laser retinopexy. A fundus examination showed numerous small, yellow, subretinal lesions in and around the macula of the right eye only. The patient had no known systemic conditions and an unremarkable family and ocular history, with 20/20 visual acuity in both eyes. Multimodal imaging findings were consistent with the newly described phenotype of benign yellow dot maculopathy. **Conclusions:** This is the second known case of unilateral benign yellow dot maculopathy. Distinct multimodal imaging findings between unilateral cases and bilateral cases may suggest differences in their etiology and manifestation.

## Introduction

Benign yellow dot maculopathy is a newly described rare macular phenotype that is asymptomatic, nonprogressive, and not associated with visual impairment.^1^ It is characterized by the presence of yellow dots in the macula, typically bilaterally. This condition can commonly be misdiagnosed as macular degeneration or another maculopathy secondary to other inherited retinal diseases, drugs, or systemic disease.^2^ At present, fewer than 50 cases have been described in the literature. The disease appears to have a sporadic or autosomal dominant inheritance pattern.^1,3-7^ No causative gene or definite etiology has been identified; thus, benign yellow dot maculopathy should be a diagnosis of exclusion, necessitating a comprehensive history, examination, and multimodal imaging that includes optical coherence tomography (OCT), OCT angiography (OCTA), and fundus autofluorescence (FAF).

To our knowledge, only 1 other case of unilateral benign yellow dot maculopathy has been published in the literature to date.^5^ Here, we present the second reported case of this unilateral condition in a middle-aged man and describe the characteristics of this finding on ancillary imaging.

## Case Report

**Figure 1.**
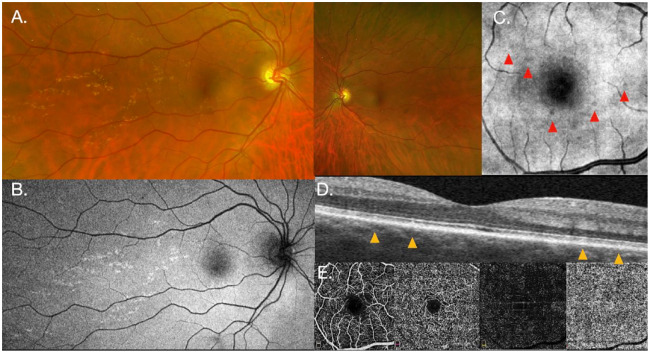
(A) Widefield imaging of the right eye (left) shows yellow subretinal lesions within and temporal to the macula. The left eye (right) appears normal and without yellow dots. (B) Fundus autofluorescence of the right eye shows hyperautofluorescent spots corresponding to the yellow dots. (C) Small nummular hyporeflective lesions are seen on near-infrared imaging. (D) Lesions corresponding to retinal pigment epithelium-ellipsoid irregularities on optical coherence tomography (OCT) of the right eye. (E) OCT angiography of the right eye is normal along the superficial and deep plexi as well as the outer retinal zone (left to right, respectively) but shows a subtle increase in choroidal vessel rarefaction at the level of the choriocapillaris (far right).

A previously healthy 42-year-old White man presented with a history of photopsias in the right eye lasting several weeks. He had no known family history of eye disease. The visual acuity (VA) was 20/20 OS. A fundus examination showed unilateral, yellow, subretinal lesions in and around the macula in the right eye only (Figure 1A), corresponding to hyperautofluorescent spots on widefield imaging (Optos) (Figure 1B). No yellow crescent around the disc or peripapillary atrophy was identified. Nummular hyporeflective lesions were seen on near-infrared imaging (Figure 1C), corresponding to retinal pigment epithelium (RPE)–ellipsoid zone (EZ) irregularities on OCT (RTVue XR Avanti, Optovue Inc) (Figure 1D). OCTA (RTVue XR Avanti, Optovue Inc) showed a very subtle increase in choroidal vessel rarefaction at the level of the choriocapillaris (Figure 1E).

An inferior horseshoe retinal tear was also noted at 6 o’clock in the right eye. The tear was treated with laser retinopexy. An examination of the anterior segment in both eyes and a retinal examination of the left eye were within normal limits. The patient was seen 6 months later for follow-up and was stable, with no change on imaging or clinical symptoms.

## Conclusions

We describe the multimodal imaging findings of what is to our knowledge the second case of unilateral benign yellow dot maculopathy. An incidental finding of unilateral small macular yellow dots in an otherwise healthy patient should be investigated and other causes carefully considered.

The differential diagnoses were thoroughly evaluated in this case, including crystalline retinopathies, familial drusen, and Gunn dots. Depending on the type of crystalline retinopathy, OCT imaging may show hyporeflective or hyperreflective foci in all retinal layers. FAF may show scattered areas of hyperautofluorescence and patchy hypoautofluorescent RPE atrophy in addition to hyperautofluorescent dots or rings with central areas of hypoautofluorescence corresponding to crystals.^2^ Familial drusen typically presents as macular and peripapillary drusen that are oriented radially in a honeycomb-like pattern. On FAF, familial drusen appears as intense autofluorescence of large drusen, while OCT imaging shows a focal or diffuse deposition of hyperreflective material between the RPE and Bruch membrane in addition to RPE elevation.^8^ Gunn dots are hyperreflective retinal structures at the level of the internal limiting membrane that are preferentially distributed in regions of thickened nerve fiber layer superior and inferior to the optic disc.^9^

In the absence of systemic signs, symptoms, or known systemic conditions, a full evaluation that included such conditions as mesangioproliferative glomerulonephritis type 2 and lipodystrophy was not performed in this case. Overall, the lack of causative agents, systemic conditions, and unremarkable family and ocular history, coupled with the anatomic size and pattern of yellow subretinal lesions and stable VA, color vision, and visual fields, made all other diagnoses unlikely.

This case and its ancillary imaging findings share features similar to the first documented case of unilateral benign yellow dot maculopathy.^5^ These features are uncharacteristic in most patients with bilateral disease. First, both patients were men, who thus far have been found to be less commonly affected by benign yellow dot maculopathy than women (33% vs 67%).^7^ Moreover, whereas in most bilateral cases the yellow dots are located around the fovea or nasal parafoveal region, both unilateral cases had additional dots temporal to the macula. Mishra et al^5^ also found RPE and EZ irregularities on OCT imaging that have typically appeared normal in patients with bilateral disease.

In the other documented case of unilateral benign yellow dot maculopathy, Goldmann kinetic perimetry and multifocal electroretinogram (ERG) were within normal limits in both eyes, although several bilateral cases were reported to have a mild-to-moderate decrease in the ERG P50 component.^3-5^ The yellow dots appeared as hyperautofluorescent dots on FAF imaging in our patient, consistent with previously described unilateral cases and bilateral cases. To date, OCTA has only been performed in 4 patients with bilateral benign yellow dot maculopathy and showed no pathologic findings in any of the cases.^4,6^ In our patient with unilateral maculopathy, OCTA showed a subtle increase in choroidal vessel rarefaction within the choriocapillaris. Thus, it is possible that there are differences in the etiology and manifestation of this condition between unilateral presentations and bilateral presentations. These differences may become more apparent and distinct as new cases of benign yellow dot maculopathy emerge.

This report highlights the essential role that multimodal imaging plays in the diagnosis of benign yellow dot maculopathy. Additional information about this disease, whose genetics and pathogenesis remain poorly understood, can be gained by clinicians, and other potential causes of yellow dot maculopathy can be ruled out.

Thought to be inherited in an autosomal dominant or sporadic pattern, previous whole exome and haplotype-sharing analyses excluded mutations in known macular dystrophy genes, including North Carolina macular dystrophy.^3^ In the future, large-scale genetic testing can assist in determining the causative genetic variants associated with benign yellow dot maculopathy.^10^ We were unable to examine the patient’s family members, resulting in a limited understanding of the potential inheritance pattern of this unilateral presentation. These are areas for future research and follow-up that could provide more comprehensive insights into this phenotype.

As more patients are diagnosed with benign yellow dot maculopathy, having a repository of rich imaging and genetic data will lead to a better understanding of this rare maculopathy.
